# Supplementary material for: Network topology mapping of chemical compounds space
Source: Sci Rep. 2024 Mar 4;14:5266. doi: 10.1038/s41598-024-54594-9 (PMC10912673; doi:10.1038/s41598-024-54594-9)
Supplement: Supplementary file 1 — Supplementary Information. [file 41598_2024_54594_MOESM1_ESM.pdf]

Supplementary of "Network topology mapping of Chemical Compounds Space"

Georgios Tsekenis,[1,2,*] Giulio Cimini,[3] Marinos Kalafatis,[4] Achille Giacometti,[2,5] Tommaso Gili,[6] and Guido Caldarelli[2,1,5,7]

[1] *Institute for Complex Systems, National Research Council, Rome, Italy*
[2] *Department of Molecular Sciences and Nanosystems (DMSN), "Ca' Foscari" University of Venice, Italy*
[3] *Physics Department and INFN, University of Rome Tor Vergata, Rome, Italy*
[4] *Department of Microbiology, University of Illinois at Urbana-Champaign, Urbana, Illinois, USA*
[5] *European Centre of Living Technologies, "Ca' Foscari" University of Venice , Venice, Italy*
[6] *IMT School for Advanced Studies Lucca, Networks unit, Lucca, Italy*
[7] *Rara Foundation - Sustainable Materials and Technologies ETS, Venice, Italy*

In this Supplementary Information we present a further analysis with methods, as well as provide more figures/results, related to the main manuscript.

I. SUPPLEMENTARY INFORMATION

A. Link density of networks

The link density of the compounds networks saturates at a finite value, while the link density for the elements networks increases, as the number of compounds in the analyzed dataset increases, as shown in Figure S1.

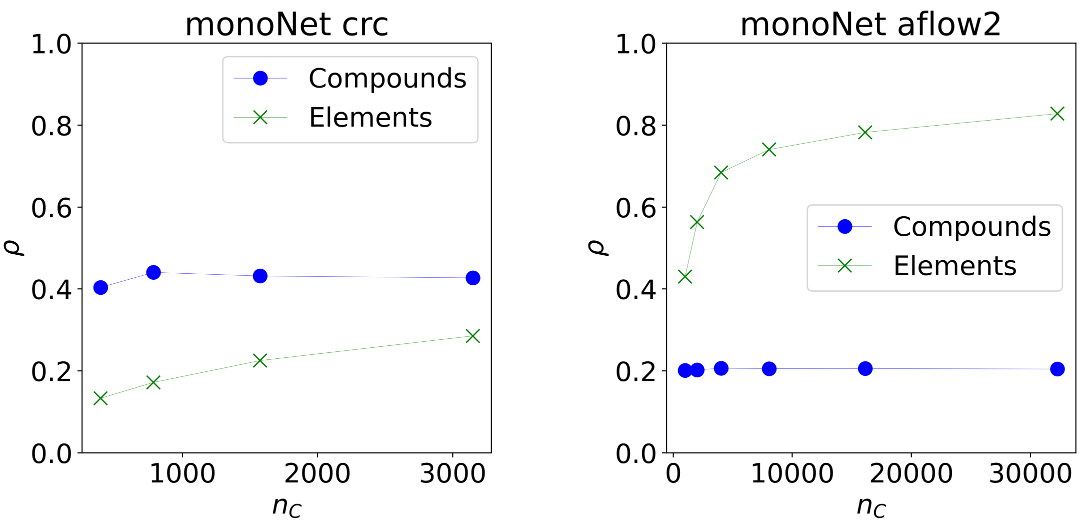

FIG. S1. Link density of the compounds, $\rho_C = L_C/(n_C(n_C - 1)/2)$ (blue dots), and elements, $\rho_E = L_E/(n_E(n_E - 1)/2)$ (green x), networks, for the CRC (left) and AFLOW (right) datasets, plotted against dataset size of the number of compounds, n_C.

B. Distributions of Normalized Degree

The distributions of normalized degrees by the total number of nodes decay faster for AFLOW than CRC. CRC has larger maximum degree relative to the maximum degree possible, as compared to AFLOW, as we show in Figure S2.

* geotsek@gmail.com

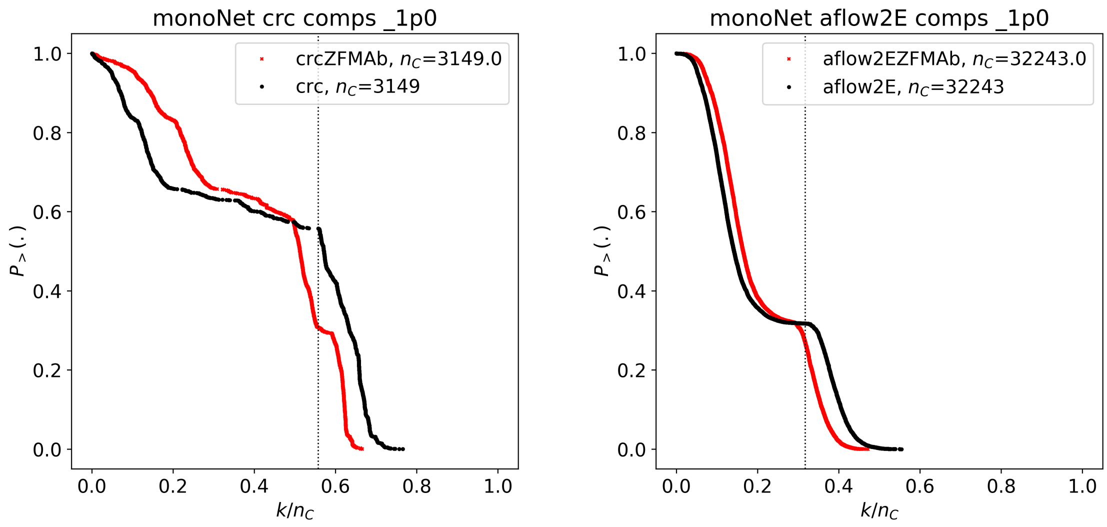

FIG. S2. Cumulative distributions of normalized degree, empirical (black dots) and calculated (red x), for the compounds of the CRC (left) and AFLOW (right) monopartite networks. As in Figure 2c,d of main, but the degrees are divided by the total number of nodes/compounds, n_C in the dataset/network.

C. Degree assortativity of bipartite networks

In the bipartite network the nearest neighbor degrees of the opposite layer are defined as

$$d_c^{nn} = \frac{1}{d_c} \sum_{e=1}^{n_E} B_{ce} d_e, \qquad d_e^{nn} = \frac{1}{d_e} \sum_{c=1}^{n_C} B_{ce} d_c \tag{1}$$

We can calculate the nearest neighbor degrees from the bipartite fitness model with a double sum

$$\tilde{d}_c^{nn} = \frac{1}{\tilde{d}_c} \sum_{e,c'} f(\delta^*, x_e^b, y_c^b) f(\delta^*, x_e^b, y_{c'}^b) \tag{2}$$

$$\tilde{d}_e^{nn} = \frac{1}{\tilde{d}_e} \sum_{c,e'} f(\delta^*, x_e^b, y_c^b) f(\delta^*, x_{e'}^b, y_c^b) \tag{3}$$

which are plotted alongside the empirical data in Fig. S3.

The degrees of the elements that are nearest neighbors to compounds, d_c^{nn}, are significantly spread so much so that they embrace both assortative and dis-assortative behaviors for the whole range of compounds degree, d_c, for both datasets, upper panels in Fig. S3. The degrees of the compounds that are nearest neighbors to elements, d_e^{nn}, vs the degrees of the elements, d_e, appear as much more disordered clouds with a strong initial assortative behavior that dissipates or tends towards weak dis-assortativty for larger d_e values, lower panels in Fig. S3. Overall assortativity is captured in on average for both the elements and compounds layers in a partial success of our model of non-interacting fermions.

D. Degree assortativity of monopartite networks

The nearest neighbor degree is defined as,

$$k_c^{nn} = \frac{1}{k_c} \sum_{c'} (A_C)_{cc'} k_{c'}, \qquad k_e^{nn} = \frac{1}{k_e} \sum_{e'} (A_E)_{ee'} k_{e'} \tag{4}$$

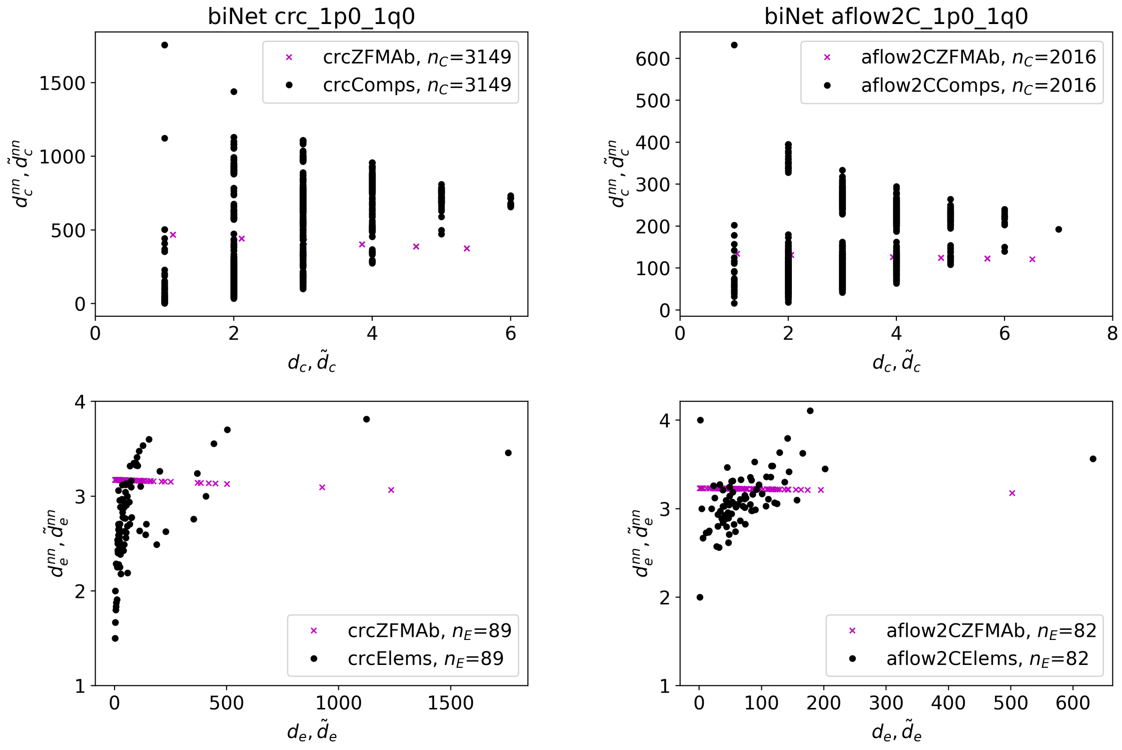

FIG. S3. Nearest neighbor degree vs degree, empirical (black dots) and calculated (purple x), for the compounds (top) and elements (bottom) layers of the CRC (left) and AFLOW (right) bipartite networks. [An AFLOW dataset with a number of compounds of $n_C = 2016$ was used for computational purposes.]

In the mono-partite network the nearest neighbor degrees are calculated as,

$$\tilde{k}_e^{nn} = \frac{1}{\tilde{k}_e} \sum_{e',e''\neq e} f(\delta_E^*, x_e^m, x_{e'}^m) f(\delta_E^*, x_{e'}^m, x_{e''}^m), \tag{5}$$

$$\tilde{k}_c^{nn} = \frac{1}{\tilde{k}_c} \sum_{c',c''\neq c)} f(\delta_C^*, y_c^m, y_{c'}^m) f(\delta_C^*, y_{c'}^m, y_{c''}^m) \tag{6}$$

The nearest neighbors degrees are plotted alongside the empirical data in Figs. S4. We find an agreement between the real networks and the fermionic fitness model as regards the dis-assortative behavior of the network of elements, Fig. S4. The degree assortativity of the compounds networks is more complicated, with a cloud for smaller degrees, and a more linear behavior at larger degrees. The cloud exhibits stronger assortative, rather than dis-assortative behavior, whereas the linear part is clearly dis-assortative. Our model has qualitatively more in common with the latter, and only captures the upper boundary of the former, in a partial success of our model of non-interacting fermions in the compounds networks.

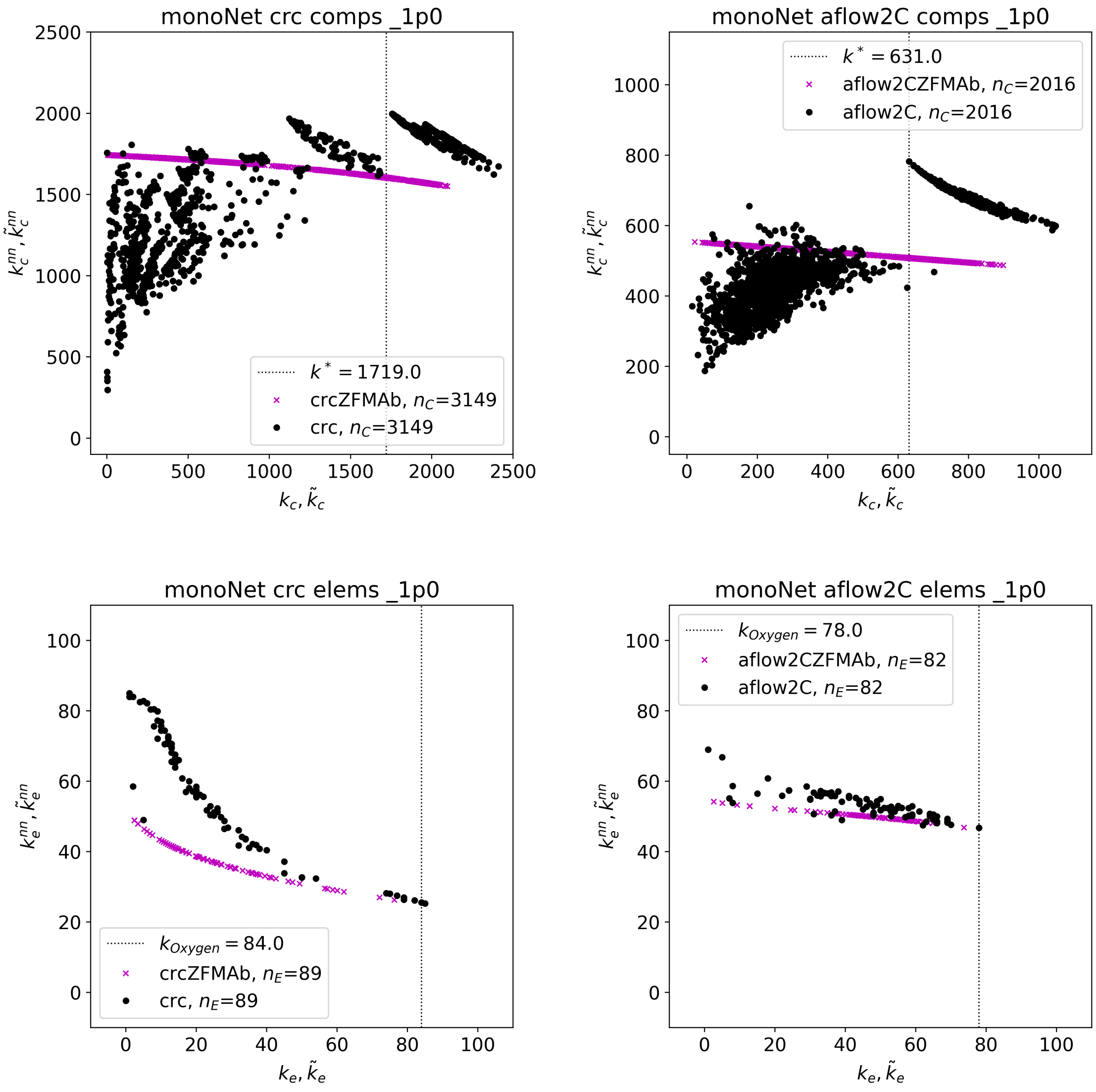

FIG. S4. Nearest neighbor degree vs degree, empirical (black dots) and calculated (purple x), for the compounds (top) and elements (bottom) of the mono-partite networks of CRC (left) and AFLOW (right). [An AFLOW dataset with a number of compounds of $n_C = 2016$ was used for computational purposes.]

E. Modeled vs empirical degree of bipartite networks

Below we show more figures with complementary results from the bipartite fitness modeling of the empirical networks. In Figure S5 we show the calculated degrees versus the empirical degrees for both layers of the bipartite networks. In Figure S6 we show the empirical and modeled degrees versus the fitness for both layers of the bipartite networks.

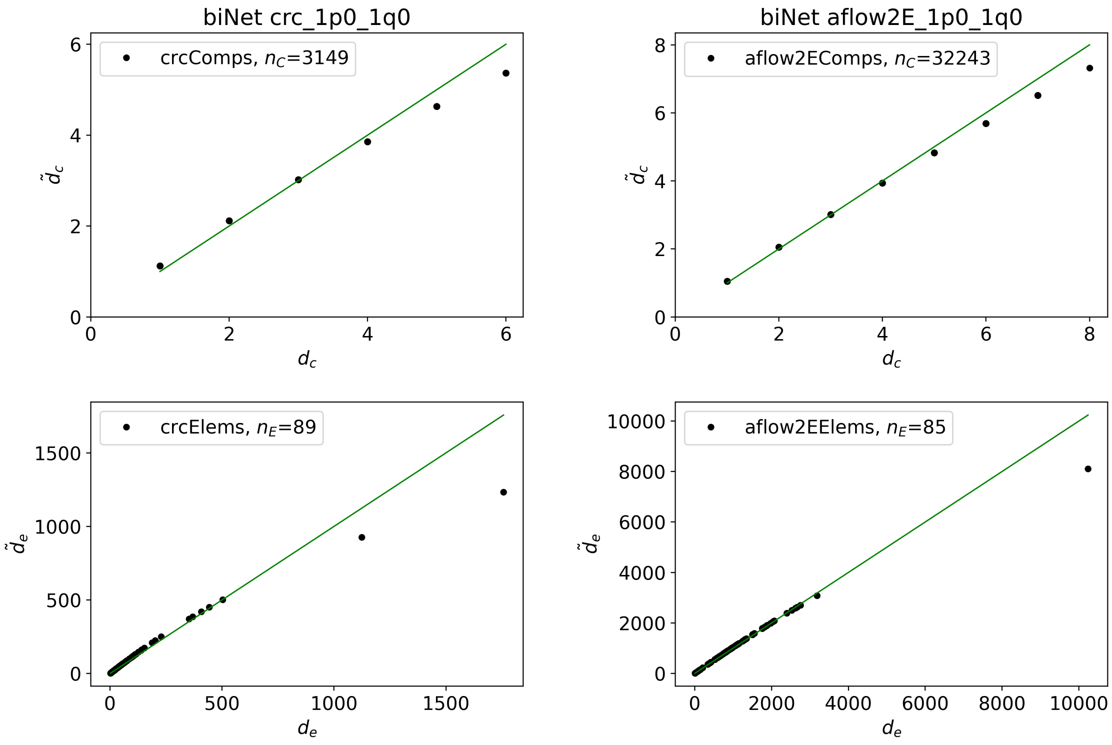

FIG. S5. The empirical degree vs the calculated degree (black dots), for the compounds (top) and elements (bottom) layers of the CRC (left), and AFLOW (right) bipartite networks. The green lines are diagonals $y = x$ as visual guides.

F. Degrees vs fitness of bipartite networks

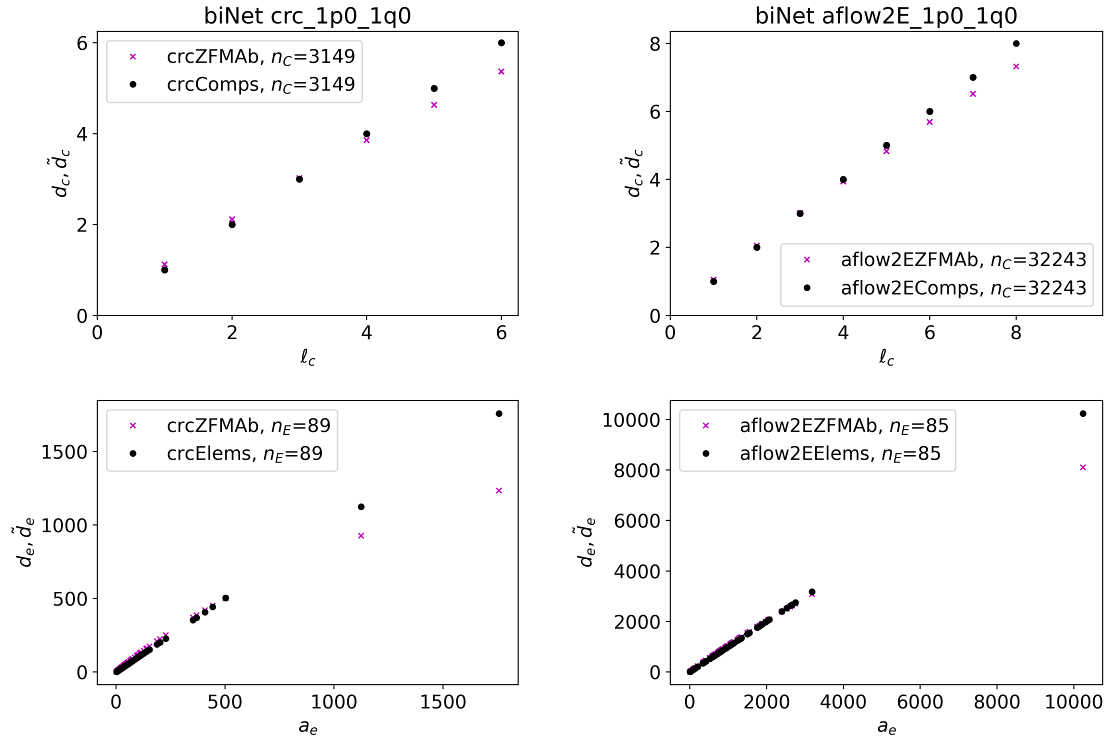

FIG. S6. The empirical degree (black dots) and calculated degree (purple x) vs fitness used in fermionic fitness model for the compounds (top) and elements (bottom) layers of the CRC (left), and AFLOW (right) bipartite networks.

G. Modeled vs empirical degree of monopartite networks

Below we show more figures with complementary results from the monopartite fitness modeling of the empirical networks. In Figure S7 we show the calculated degrees versus the empirical degrees for both monopartite networks. In Figure S8 we show the empirical and modeled degrees versus the fitness for both monopartite networks.

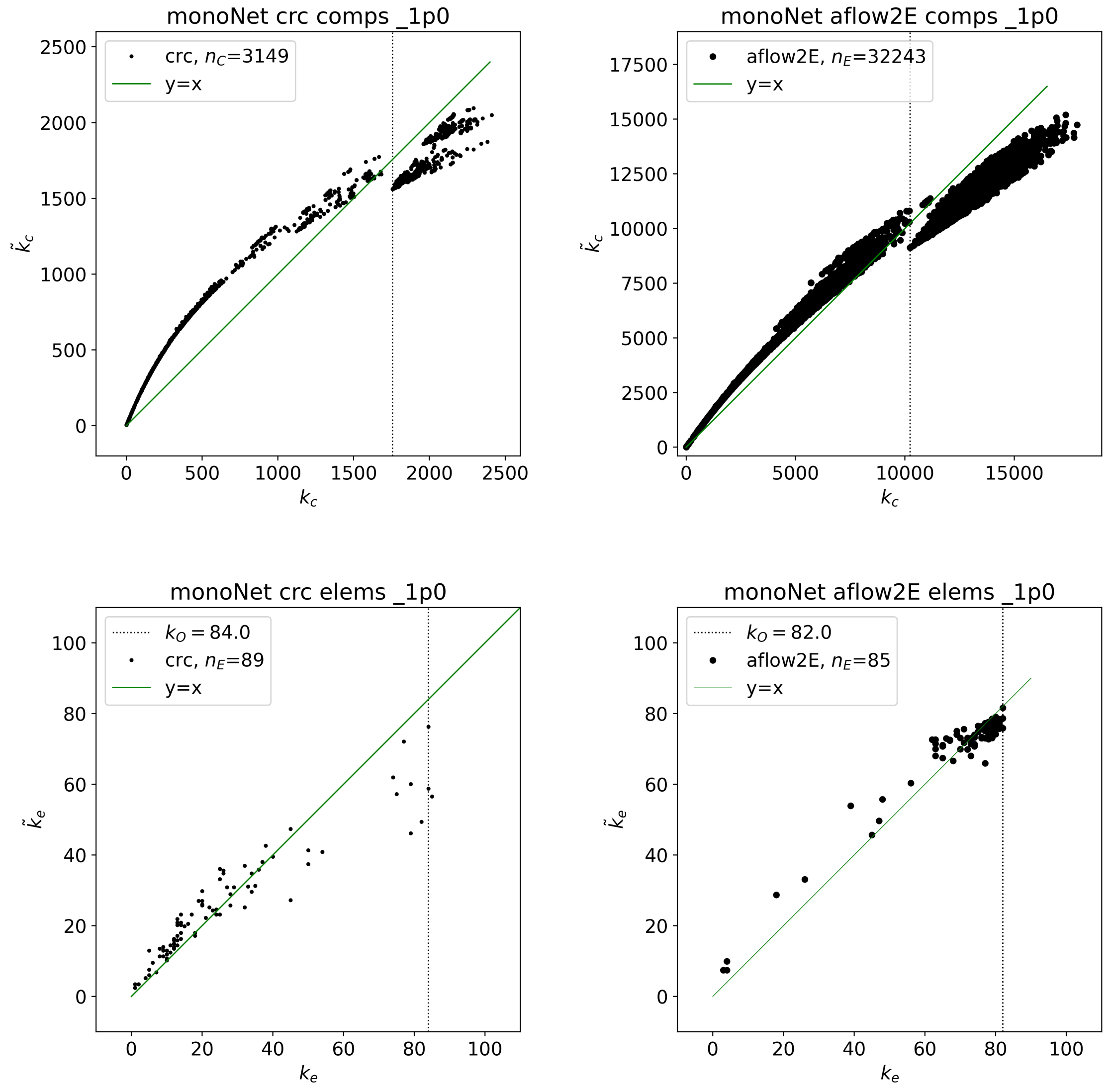

FIG. S7. The empirical degree vs the simulated degree (black dots) for the compounds (top) and elements (bottom) monopartite networks of CRC (left) and AFLOW (right). The green lines are diagonals $y = x$ as visual guides.

H. Degrees vs fitness of monopartite networks

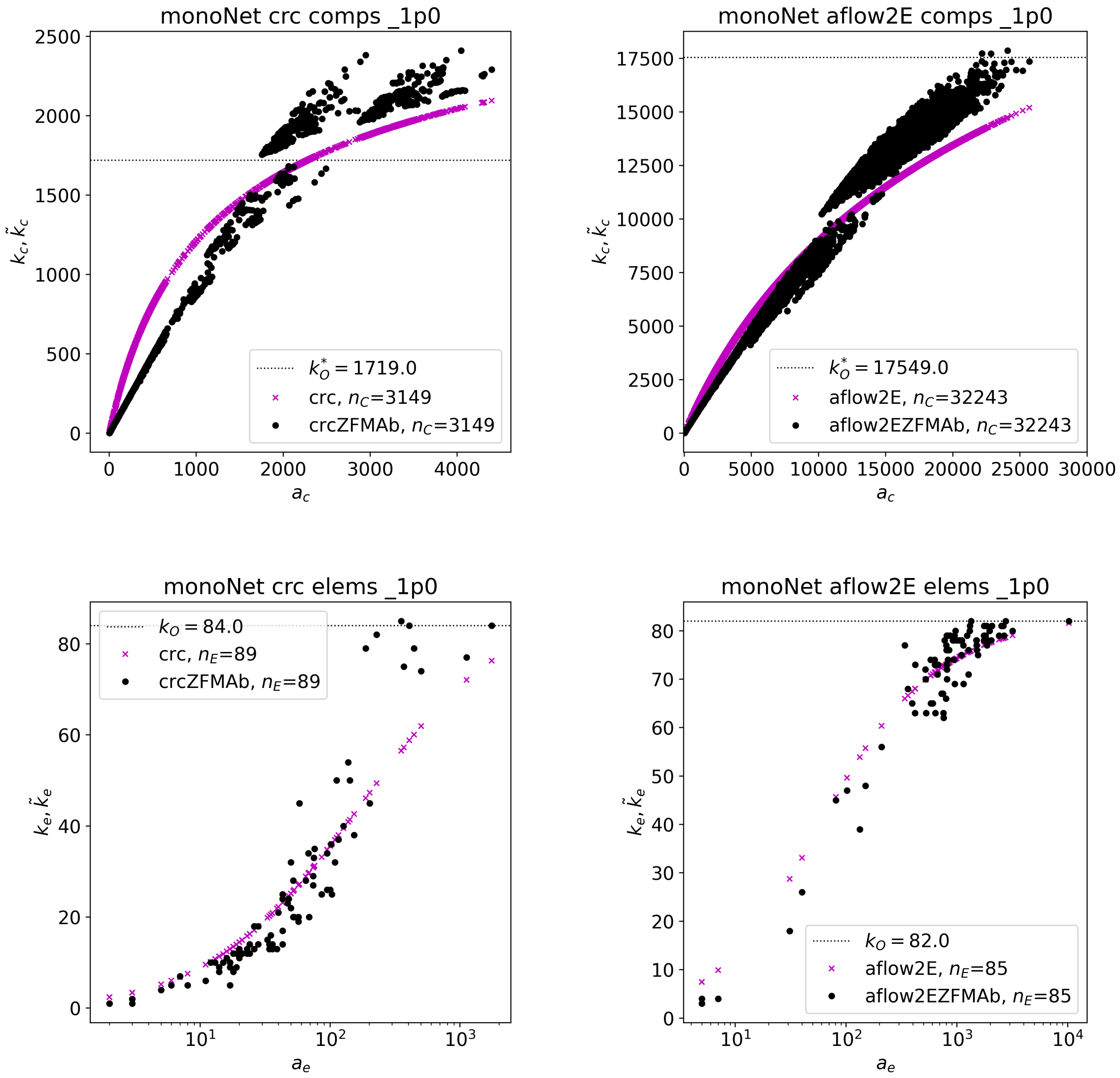

FIG. S8. The empirical degree (black dots) and simulated degree (purple x) vs fitness for the compounds (top) and elements (bottom) mono-partite networks of CRC (left) and AFLOW (right).
